# Supplementary material for: Evaluation of a comprehensive health check offered to frontline health workers in Zimbabwe
Source: PLOS Glob Public Health. 2024 Jan 8;4(1):e0002328. doi: 10.1371/journal.pgph.0002328 (PMC10773946; doi:10.1371/journal.pgph.0002328)
Supplement: S1 Table — (DOCX) [file pgph.0002328.s002.docx]

*S1 Table: Process evaluation qualitative reports (Themes, codes and supporting quotations from workshops)*

| **Themes** | **Codes** | **Key quotes** |
| --- | --- | --- |
| Contextual factors that made the service appealing and acceptable | Financial constraints hindering uptake of health services | “I was referred to get my eyes checked. Of which I wasn’t able to do it because of financial problems considering the money that we are paid and looking at the situations in our families… and how expensive access to health is…that there is nothing you can do…there is no special treatment given to staff from health sector your earnings are supposed to assist you yet the earnings are not sufficient.” (Facility manager, Male, 57) |
|  | Under resourced health system | “…considering our health system… at times people might want to go and get treated but if we don’t have glucose stripes it means one won’t be able to be tested for sugar.” (Nurse, Female, 30) |
|  |  | “It was poor management of BP that pushed me……I was diagnosed in 1998 and I was not taking my medication very well. I was not taking them as per prescribed combination.” (Youth Mentor, Male, 52) |
|  | Poor health seeking behaviour | “So, I have seen as health workers we tend to put a blind eye when it comes to our health and say we will see. Sometimes its fear, but sometimes is the lack of interest and ignorance.” (Nurse, Female, 30) |
|  |  | “Health care workers are always busy nursing patients and they tend to forget themselves. The intervention made it possible for them to know their health status.” (Intervention staff, Nurse, Female, 47) |
|  | Free of charge | “It would actually be a 10/10 (score) and it was free to get a diagnosis looking at the fact that I can’t afford even the consultation on my own.” (Nurse, female, 27) |
|  |  | Yes, in the past we did not pay here but this time if you want to be consulted or anything they no longer consult for free, that’s why we are happy for your services”. (Facility manager, male, 61) |
|  | Accessible | “Then the other thing is that you are coming to us…at times we don’t create time to go for just a health check-up. We just then go for testing when we are already sick so I think it’s helping us a lot…” (Nurse, Female, 30) |
|  | Comprehensive – a one stop shop | “I would say it was quite an interesting experience. You know that moment when you get everything in one package. Getting your blood pressure checked, your eyesight, screened for COVID and even checked up on your mental health… having a one stop shop. It was a beautiful experience if I might say.” (Nurse, female, 31) |
|  |  | “When implementing the intervention, I liked screening health workers for different diseases such as BP, diabetes etc.” (Intervention staff, Nurse, Female, 47) |
|  |  | “For me, it was a total package because they checked my sugar levels, BP, COVID test and also HIV.” (Accountant, male 48) |
|  |  | “It was fine because at least I got screened for a lot of things my eyesight, my mental health, vital signs…” (Nurse, female, 34) |
|  |  | “They would do everything they came to do … tests, depending of what you wanted to be done and they said they would do follow ups for those that would have tested positive for different disease and they would assist on how they could be further helped. I was screened for BP. I was screened for diabetes. I was screened up to my blood. Then the tests results indicated that my BP was very high.” (Security guard, male, 42) |
|  |  | “My temperature was checked, BP, height, weight and visual acuity and then a bit of some information on how to deal with stress management, and I also got an HIV test, my blood sugar was checked and also some health education on diet.” (Nurse, female, 30) |
|  | High quality screening | “I also liked the HBA1C test because it’s different from getting tested (with a glucometer)… it’s a useful screening tool that can actually tell if someone is diabetic or not.” (Sister-in-charge, female, 39) |
|  | Efficiency | “The service was good, it was professional. The way they did their operations, everything was good, even when I was tested it didn’t take time, I arrived and got service immediately.” (Primary Counsellor, Female, 41) |
|  |  | Those that I consulted would just say the BP is okay. But they would not explain clearly so much that I would be left hanging. So now that I heard that the program is back (ICAROZ) I am pleased and I actually came here running.” (Security guard, male, 42) |
|  |  | “I did not wait that long before getting assisted, I was actually assisted in a good way” (RGN, Female, 27) |
|  | Friendly staff | “The service is good. We feel at ease coming here than the staff clinic because we know our privacy will be respected.” (Nurse, male 33) |
|  |  | “The providers are very friendly…that makes one feel like opening up.” (Dental Assistant, Female, 42) |
|  | Private and confidential | “When I visited the tents, the staff were polite, and they did their testing privately and confidentially” (Nurse, Female, 29) |
|  |  | “Haa it was good enough because no one could see what you were being tested for… you just get your results and in confidence…” (Primary Counsellor, Male, 29) |
|  |  | “They just give you your results and say here they are so you could just change and if you want but they are hidden with numbers and not names so it’s good”. (Counsellor, Male, 29) |
|  |  | “Like here, they don’t know us right and we don’t know them right, so everyone is free to come. Unlike for me to go to the staff clinic, I will think this sister might tell people about me. So, I might not go even when I know they are offering that service. If you look at the number of people coming here and those going to the awareness clinic here…” (Dental Assistant, Female, 42) |
|  |  | “Confidentiality is there because a person goes into a separate room without people and they discuss their issues there, no one will know what they are talking about, so, confidentiality is there”. (Nurse, Female, 34) |
|  |  | “We don’t even know you (ICAROZ staff), even if I do an HIV test and its positive, there is an issue of confidentiality. There is no way you would go spreading the news that I am positive.” (Facility manager, Female, 39) |
|  |  | “The services were so fantastic; they will also help to improve disease management and prevention as far as health workers are concerned.” (Counsellor, Male, 29) |
|  |  | “All health workers in hospitals and clinics where we conducted the study were happy to be screened for various conditions by unfamiliar people. This made them feel comfortable and open up. Despite conducting Wellness clinics at their workplaces, they are not comfortable because they have no assurance of privacy and confidentiality.” (Intervention staff, Nurse, Female, 47) |
| Perceived benefits/ positive attributes of the services | Improved health management and health outcomes | “But I was not too surprised that my BP was high because there is a time it was border-line but I was really happy to know because BP is a silent killer. Now that I know I can control my salt intake and have regular BP checks.” (Midwife, Female, 59) |
|  |  | “After that we got inside where they did tests on BP, sugar. That is what I still remember and my BP was found to be high. And they said go to the doctor. If the BP is high by then, I will have to start medication.” (TB TPT Champion, Female, 42) |
|  |  | “I also got a test for blood sugar but they said it was a bit high. They recommended me to go and see the doctor. …. I got to find out about it on the day I came for screening, I had never been diagnosed with diabetes before. (Nurse, Female, 30) |
|  |  | “The services are very good because when I met them, I was a bit depressed and I was helped, I managed to get counselling, because the time I was helped, I was suicidal. Mental health was relevant to me because I was having problems. I had already given up…now I’m feeling better.” (Security, Female, 38) |
|  | Prioritizing addressing health workers' health needs | “As, health workers, I can say everything was valuable…because we take care of the patients, and no one is taking care of us.” (Dental Assistant, Female, 42) |
|  |  | “Yes, because you know I always ask this question, who will nurse the nurse? No one… but we also need that. Most of the times when the nurse gets sick the problem is that the condition is discovered when it’s now way too late.” (Nurse, Female, 31) |
|  |  | “I feel that it’s crucial to always get screened because sometimes we might just be helping others while our health is deteriorating.” (Nurse, Female, 34) |
|  |  | “Looking at health care workers we meet a lot of patients and treat them for various conditions. But for a health care worker to go somewhere to seek medical attention it might be difficult.” ((TB TPT Champion, Female, 42) |
|  |  | “Others were saying what is this about? We can’t spend time being weighed … waiting around to get your height measured….” (Primary Counsellor, Male, 29) |
| Barriers to service uptake and linkage to care | Lack of interest | “Mmm, time, waiting time is, is, is a lot. As staff we are inviting each other at once, inviting each other to go, about 10 of us at once so you will see as though… So, I waited about 10 minutes and I felt I was being delayed then I decided to go back. (Dental Assistant, Female, 42) |
|  |  | I wasn't happy that the healthcare workers with high BP and raised blood glucose were refusing referral letters. They dismissed the referrals saying that we shouldn't worry they would be fine and would conduct daily checks on their own... I felt sad because I wasn't expecting that from healthcare workers. They render medical services to others but unfortunately, they do not want to be on the receiving end yet they are well informed. |
|  | Duration of service provision | “I think the rooms are few, like…in the first room, where another person is having their OBS done and you are having a questionnaire done, there are other people who want to…who have big ears -who ease drop. So, maybe they were supposed to say, those who are being asked questions should be done privately.” (Dental Assistant, Female, 42) |
|  | Privacy | “One might have been referred but the money to do further tests might not be available. Because you might arrive there and you are told of a huge amount and yet you don’t have the money.” (Midwife, Female, 44) |
|  | Financial constraints | “I was told that I should see a doctor for my eyes, but where do I get the money and where would I get the money for spectacles.?” (Nurse-aide, Female, 44) |
|  |  | “So, I just thought I should stay like that, you see…the reason being that I cannot afford. Yes, I cannot afford, because of huge family responsibilities and it’s now a burden on my end, I have no solution.” (Nurse, Male 61) |
|  |  | “Considering our remuneration, you can be engaged and referred to go see a doctor but because of finance maybe you have no money for transport to go there.”  (Nurse, Female, 30) |
|  |  | “What they did not like about the intervention is they would say you are just providing testing services without treatment and we do not have the money to get treatment after being diagnosed” (Intervention staff, Nurse, Male, 39) |
|  |  | “I was referred to get my eyes checked. Of which I wasn’t able to do it because of financial problems considering money and the money that we are paid looking at the situations in our families… and how expensive health is…that there is nothing you can do…there is no special treatment given to staff from health sector your earnings are supposed to assist you, yet the earnings are not sufficient.” (Nurse, Male, 57) |
|  |  | “In our institutions on the issue on drugs they don’t have, they will write you a prescription to go and buy from the pharmacy. Then you will see that the drug that you need to buy it’s expensive. So we have people with conditions such as BP who are stroking because of that. They will say, ‘I failed to get the drug I wanted, I couldn’t get nifedipine. At the same time I didn’t have money to go and buy somewhere else so I just stopped taking’, so they will tell you the truth that I stopped taking it.” (Nurse, Male, 61) |
|  |  | “Like at my age… at my age it’s impossible to be diagnosed with BP. I am 30, and at my clinic I actually told them I am still young, and if I start now being diagnosed with BP what more when I get old? (Primary Counsellor, Female, 30) |
|  | Denial | “I got a little scared because honestly. I wasn’t prepared and it came to me as a shock, and I was a little scared... Because thinking that I was diagnosed with diabetes at this age, ah no. (Nurse, Female, 29) |
|  |  | “Someone would have not accepted the diagnosed condition that it is possible in such a situation. Acceptance is the problem is.” (Data Entry Clerk, Male, 35) |
|  |  | “People are afraid the diagnosis because they think its end of life, they are in denial.” (Youth Mentor, Male, 52) |
|  |  | “Sometimes you might finish late at work and you might think twice to go and see a doctor after work. Then you would then have to wait for off days. But maybe during this time of waiting the condition is worsening and damaging. (Security Guard, Male, 36) |
|  | Time constraints | “You will be referred to go somewhere and right now you know our work is hard, we are short-staffed. You cannot say I can’t come to work; I am at a queue to see the doctor; you will see it better not to go… they are slow to serve you. And the time that the doctor comes you would be supposed to be at work. So, you end up going back before the doctor consults.” (Midwife, Female, 44) |
|  |  | “So, the time that I will waste standing in a queue especially at the general health care facilities. I decide I would rather let it be.” (Primary Counsellor, Female, 30) |
|  |  | “If I don’t come to work for a day, that day will be removed from my salary because I am paid according to hours worked. And going to a public health institution there is no way I will receive service on a weekend. (Primary Counsellor, Female, 46) |
|  |  | “Such a good service needs to expand (e.g., include STI, treatment services, training sessions on infection prevention) and to be well-staffed to meet our demand” (Nurse, female, 29) |
| Recommendations on service improvement | Expansion of services | “Maybe what I can say is that, if you can have more screening tools to include TB. Maybe to have a mobile x-ray machine which you can use to screen there and there.” (Nurse, female, 30) |
|  |  | “Yeah, I was talking to them saying why don’t you do, family planning services, I would have had one today” (Nurse, Female, 34) |
|  |  | “I think you need to do it maybe this after every 6 months or yearly.” **(**Facility manager, Female, 39) |
|  |  | “Given a chance to provide this intervention again, I would recommend that we invest in extending the reach and offer the services to health workers who work in remote (rural) areas as they could have also benefited from the services such as the HBA1C testing which is not easily accessible and is expensive.” (Intervention staff, Research assistant, Female, 30). |
|  | Repeat service provision | “So, it all went well, we hope you will keep coming back.” (Ambulance driver, Male, 48) |
|  |  | “I would recommend you make two visits a year at each facility.” (Casualty nurse, Male, 33) |
|  |  | “I think you have to do this on a quarterly basis so that you can be able to cover all health care workers.” (Primary Counsellor, Male, 29) |
|  |  | “Let’s say you have met me and you have identified I have such a problem. You are supposed to have a direct referral where I can go and get help for free.” (Facility manager, Male, 61) |
|  | Free referral services | “I feel like if the service is free and does not need money someone might quickly accept it. Like if you have other clinics and you refer us to these clinics to receive help. That might result in people quickly accepting than when am diagnosed with BP then you tell me to go and see a doctor, I might not have the money to go and see the doctor. I feel like it helps if we receive the full package here.” (TB TPT Champion, Female, 42) |
|  |  | “You can sort of motivate us that even through hardships, we have facilities, or we have these incentives or we have doctors that can see us for free.” (Security Guard, Male, 44) |
|  |  | “I feel like if the service is free and does not need money someone might quickly accept it. Like if you have other clinics and you refer us to these clinics to receive help. That might result in people quickly accepting than when am diagnosed with BP then you tell me to go and see a doctor, I might not have the money to go and see the doctor. I feel like it helps if we receive the full package here.” (TB TPT Champion, Female, 42) |
|  |  | “Maybe on your team if you can have a doctor so that when you do your referrals, if someone is comfortable, they can then proceed into the doctor’s office. Then they immediately see the doctor.” (Nurse, Female, 30) |
|  | Hiring doctors and specialists on the team | “There should be a doctor at your workplace so that you refer us firstly to your doctor then we go and get examined by your doctor. Then the whole system will just be done and be through at once. I have been tested I have DM, I have diabetes, I have HIV, and then everything is just managed there. If am to go any further I am going further with the medication I would have been given.” (Primary counsellor, Male, 47) |
|  |  | “Future studies should have a team that includes a medical doctor, professional counsellors, anti-hypertensive drugs and oral diabetes drugs to do screening, diagnosis and initiate treatment to those eligible so that we can have fit healthcare workers.” (Intervention staff, Nurse, Female, 47). |
|  | Provision of free treatment and medication | “If you test someone and see that they need medication…it’s better that you give him the medication yourself… so that there is no gap… Also, to give eyeglasses to those that have visual problems. They would want the glasses because the eye specialists are very expensive, they cost around $200.” (Midwife, Female, 44) |
|  |  | “The health care workers suggested that we should have a doctor on site so that after screening they can have consultation with him/her. They also suggested that we supply anti-hypertensive and anti-diabetes drugs.” (Intervention staff, Research assistant, 36) |
