# Supplementary material for: Evaluation of a comprehensive health check offered to frontline health workers in Zimbabwe
Source: PLOS Glob Public Health. 2024 Jan 8;4(1):e0002328. doi: 10.1371/journal.pgph.0002328 (PMC10773946; doi:10.1371/journal.pgph.0002328)
Supplement: Attachment — Submitted filename: Review of Manuscript Number PGPH-23-01432.pdf [file pgph.0002328.s004.pdf]

Manuscript Number PGPH-D-23-01432

Reviewer Comments

1. This is an original well-designed, relevant, timely and well-reported study on the implementation and uptake of a health check service for Zimbabwe's health care workers during the COVID-19 pandemic, 2020 – 2022. The health care deficit addressed is explained clearly.
The best attributes of this paper are the clearly explained and justified methodology and approaches used during the evaluation. In the results, the detection of undiagnosed hypertension and diabetes was/is a very valuable aspect of this study and any future follow up programmes. Even if treatment is expensive or not available, awareness is important and life style changes can make a different in some cases.

2. However, there is room for improvement in the following:

2.1 **Legal, Ethical and data security issues**. Lines 249 – 255 summarise the ethical approvals.

- 2.1.1 Other than the informed consent, the other ethical, legal and data protection issues that arise/arose in this study and the mitigation measures taken are unclear.
- 2.1.2 What happens (ed) to the recordings and transcripts? How did the study manage these so as to guarantee security, confidentiality, privacy and security during and after the study?
- 2.1.3 **The authors are multi**-sited and this implies that data may have been transmitted across national territories. If this was/is the case, what mechanisms were employed to share data across boundaries and institutions and what measures were used to ensure data security?

There are some small points for clarification, *viz*:

2.2 Lines 242 – 246 – in the methods section on coding, the study identified deductive and inductive themes. However, the specific themes identified for each category and how they were used to organise the presentation of results is unclear.

2.3 The target was health workers. It is unclear why and how police and army personnel became part of the study as reported in the results (lines 271 -274).

2.4 The study reports on age, sex distribution of clients (lines 270 – 271) indicating that 79% of them were female. How do the proportions reported in the study compare to those in the total national or provincial health workforce? This issue comes up again later in lines 444 -451 (page 25) where it states that 76% of Zimbabwe's nurses are female. The 3% difference suggests that the proportions in the study come close to those in the national workforce. It is surprising that the stratified numbers/percentages in the total workforce are not available from the relevant ministry.

2.5 Reference is needed for the claim made on "… low sensitivity in African populations …" at page 27 lines 494 – 496.

3. Further attention is needed to address a few errors of grammar.
4. One would expect spatial variation in the health conditions, experiences and service expectations among the clients; remote rural centres compared to the metropolitan areas for instance. If the study did not have scope to reflect on this, then this could also be recommended as a candidate for inclusion in future studies.

Recommended for publication with minor corrections. END
