# Supplementary material for: Evaluation of a comprehensive health check offered to frontline health workers in Zimbabwe
Source: PLOS Glob Public Health. 2024 Jan 8;4(1):e0002328. doi: 10.1371/journal.pgph.0002328 (PMC10773946; doi:10.1371/journal.pgph.0002328)
Supplement: Attachment — Submitted filename: Response to reviewers _ PGPH-23-01432_Final.docx [file pgph.0002328.s005.docx]

The Academic Editor
PLOS Global Public Health

Re: PGPH-D-23-01432

Thank you for considering our publication entitled *“Evaluation of a comprehensive health check offered to frontline health workers in Zimbabwe”.* Thank you also to the reviewers for their time and constructive comments. We have addressed the points raised point-by-point below. We believe this has improved the quality of the manuscript.

Journal Requirements:

1. Please include a complete copy of PLOS’ questionnaire on inclusivity in global research in your revised manuscript. Our policy for research in this area aims to improve transparency in the reporting of research performed outside of researchers’ own country or community. The policy applies to researchers who have travelled to a different country to conduct research, research with Indigenous populations or their lands, and research on cultural artefacts. The questionnaire can also be requested at the journal’s discretion for any other submissions, even if these conditions are not met. Please find more information on the policy and a link to download a blank copy of the questionnaire here: <https://journals.plos.org/globalpublichealth/s/best-practices-in-research-reporting>. Please upload a completed version of your questionnaire as Supporting Information when you resubmit your manuscript.

*The questionnaire has been attached. Thank you.*

2. Please review your reference list to ensure that it is complete and correct. If you have cited papers that have been retracted, please include the rationale for doing so in the manuscript text, or remove these references and replace them with relevant current references. Any changes to the reference list should be mentioned in the rebuttal letter that accompanies your revised manuscript. If you need to cite a retracted article, indicate the article’s retracted status in the References list and also include a citation and full reference for the retraction notice.

*Noted with thanks.*

Reviewer #1

3. This is an original well-designed, relevant, timely and well-reported study on the implementation and uptake of a health check service for Zimbabwe’s health care workers during the COVID-19 pandemic, 2020 – 2022. The health care deficit addressed is explained clearly.

The best attributes of this paper are the clearly explained and justified methodology and approaches used during the evaluation. In the results, the detection of undiagnosed hypertension and diabetes was/is a very valuable aspect of this study and any future follow up programmes. Even if treatment is expensive or not available, awareness is important and life style changes can make a different in some cases.

*Thank you.*

4. However, there is room for improvement in the following:
   4.1 **Legal, Ethical and data security issues**. Lines 249 – 255 summarise the ethical approvals.
       4.1.1 Other than the informed consent, the other ethical, legal and data protection issues that arise/arose in this study and the mitigation measures taken are unclear.

*Thank you for your comment. The institutional and national IRB approvals in Zimbabwe included appraisal of both ethical requirements and legal issues required by Zimbabwean law. This is mentioned in lines 275 – 281: “Ethical approval was granted by the Medical Research Council of Zimbabwe (MRCZ/A/2627), and the London School of Hygiene & Tropical Medicine ethics committee (22514) prior to participant recruitment.”*

*Text has been added under Ethical review. Line 281 which reads: “The UK and Zimbabwean data protection regulations were adhered to”.*

*Other data security issues are addressed as part of the response to comment 4.1.2 below.*

4.1.2 What happens (ed) to the recordings and transcripts? How did the study manage these so as to guarantee security, confidentiality, privacy and security during and after the study?

*Text has been added describing how recordings were handled in compliance with data protection principles. Lines 247 – 251 now reads;*

*“Recordings from interviews were stored on an encrypted hard drive. Transcriptions, in which participant identifiable information had been removed, were uploaded to a secure cloud server accessible to authorized study staff only. Following checking of transcriptions for accuracy, audio recordings were deleted.”*

4.1.3 **The authors are multi**-sited and this implies that data may have been transmitted across national territories. If this was/is the case, what mechanisms were employed to share data across boundaries and institutions and what measures were used to ensure data security?

*All analysis was conducted in Zimbabwe. There was no data sharing across national boundaries.*

There are some small points for clarification, *viz*:

4.2 Lines 242 – 246 – in the methods section on coding, the study identified deductive and inductive themes. However, the specific themes identified for each category and how they were used to organise the presentation of results is unclear.

*Thank you for this comment, we have addressed it by providing more detail in the methods section on the coding and analysis process. We mentioned the specific themes identified for each category and how we organized them (see lines 257-273).*

4.3 The target was health workers. It is unclear why and how police and army personnel became part of the study as reported in the results (lines 271 -274).

*In large hospitals in Zimbabwe, there is a permanent police base for security purposes and processing of any relevant documentation reserved for their authority. Furthermore, during the COVID-19 pandemic, more police and army personnel with health-related qualifications were deployed to health facilities to provide support to the strained health services.*

*As mentioned in the manuscript, “All staff who were working at a health facility where the service was offered were eligible, regardless of their role and employer” (lines 148 -149). This meant that police and army personnel working at health facilities were included in the service.*

4.4 The study reports on age, sex distribution of clients (lines 270 – 271) indicating that 79% of them were female. How do the proportions reported in the study compare to those in the total national or provincial health workforce? This issue comes up again later in lines 444 -451 (page 25) where it states that 76% of Zimbabwe’s nurses are female. The 3% difference suggests that the proportions in the study come close to those in the national workforce. It is surprising that the stratified numbers/percentages in the total workforce are not available from the relevant ministry.

*Thank you for highlighting this. We have added a statement to make the comparison clear on line 523-524. It now reads as;*

*“In Zimbabwe 76% of nurses are women,(42) almost equal to the proportion of women who accessed the health screening services.”*

*Regarding availability of sex-stratified numbers of healthcare workers in each cadre in the total workforce, there are no publicly available estimates. Whilst these data may be available at each institution, or at national level, we did not include data access requests for this information in our protocol or ethical approvals.*

4.5 Reference is needed for the claim made on “… low sensitivity in African populations …” at page 27 lines 494 – 496.

*References have been added on line 564. Thank you.*

5. Further attention is needed to address a few errors of grammar.

*Noted with thanks.*

6. One would expect spatial variation in the health conditions, experiences and service expectations among the clients; remote rural centres compared to the metropolitan areas for instance. If the study did not have scope to reflect on this, then this could also be recommended as a candidate for inclusion in future studies.

*A great idea which can be considered for future studies. Noted with thanks.*

**Reviewer #2**

This is a good paper about a relevant and important topic especially given how the well-being of health workers is often overlooked. Please see below my suggestions to help strengthen the manuscript.

*Thank you*

INTRODUCTION
The authors may want to include literature that shows the conditions/ problems that typically affect health workers to justify the need for health checks.

*Literature has been included to paragraph 4 (Lines 96 – 102) which reads;*

*“The limited data available suggest that undiagnosed chronic diseases such as diabetes, anaemia, and hypertension are highly prevalent in Zimbabwe. These conditions likely affect healthcare workers to a similar extent as the rest of the population. Diagnosing and treating chronic conditions has the potential to enhance healthcare workers’ well-being. In the context of COVID-19 this is even more important because undiagnosed and poorly controlled chronic conditions are risk factors for severe COVID-19.”*

METHODS
I suggest placing the “Health check” section [line 166-189] before the “Service setup section” [line 144-162]. This will help the manuscript read better by first describing the intervention and then how it Is set up.

*Thank you for this suggestion. However, in our manuscript we intended to outline how we provided a health check service in such a way as to illustrate how this might also be done in other settings. By placing “Service setup” before “Health check”, we believe it will help readers appreciate the principles of how the health check was configured – for example how the workstation would be prepared and roles delegation among our staff, taking into consideration issues of confidentiality and infection prevention and control – before introducing the specific services offered. The services themselves are more flexible, as shown during our study where additional services were added during the study period, and could be different in different settings, whilst adhering to the same overall principles of service provision.*

RESULTS
• Not sure if some of the referrals were followed up to see the uptake of referral service? It would be beneficial to know the proportion of those who utilized the referral services.

*Thank you for acknowledging the importance of going further discussing the referral services. These findings are part of a separate manuscript which is currently under peer-review at a different journal.*

• In the methodology, the authors mention that they interviewed service providers (assuming these were the delivering agents on the intervention). However, in the results section, their views are not present or have not been distinctly labelled. It would strengthen the manuscripts to also include their experiences delivering the intervention and improvements that might need to be made to the intervention.
• It would be beneficial to hear their findings on sustainability from the service providers (e.g., time, workload).

*Thank you for this suggestion. We have now included key quotes (both in-text and in the supplementary table) that highlight the experiences and views of the service providers. The expressed views include sentiments on what they liked about the intervention, what struck out to them about health worker uptake of services, and recommendations for future interventions.*

*Recorded insights from the intervention staff (service providers were added to Supplementary 2 table). Findings from the intervention staff were also added to the results section as text and as quotes (Lines 370 - 479).*

DISCUSSION
The authors did not discuss how their intervention compares to other health workers/ frontline worker interventions that were implemented whether before or during the COVID outbreak.

*Thank you for this suggestion. We have added some text in the discussion explaining that the service was an innovation. Line 575 -578 now reads as:*

*“Whilst there were many interventions, globally, that provided SARS-CoV-2 testing or mental health support for healthcare workers during the COVID-19 pandemic, we are not aware of any other comprehensive health services developed for health workers during this period”*

*.*

*In addition, as part of a related manuscript, we conducted a literature search to identify studies reporting multimorbidity or key chronic conditions among healthcare workers in Africa, since January 2003. This did not identify any studies providing comprehensive health services to healthcare workers.*
