# Supplementary material for: Evaluation of a comprehensive health check offered to frontline health workers in Zimbabwe
Source: PLOS Glob Public Health. 2024 Jan 8;4(1):e0002328. doi: 10.1371/journal.pgph.0002328 (PMC10773946; doi:10.1371/journal.pgph.0002328)
Supplement: Attachment — Submitted filename: Response to reviewers _ PGPH-23-01432_Final_2.docx [file pgph.0002328.s006.docx]

The Academic Editor
PLOS Global Public Health

Re: PGPH-D-23-01432

Thank you again for considering our publication entitled *“Evaluation of a comprehensive health check offered to frontline health workers in Zimbabwe”.* Thank you also for the constructive comments. We have addressed the issues raised in our point-by-point response below. We believe this has improved the quality of the manuscript.

1. Currently, the manuscript does not include a discussion on potential sources of bias. Please address this in the second revised version.

*Thank you for the comment. We have added a discussion of potential sources of bias under the discussion section. Lines 559 – 566 now reads:*

*“We acknowledge several limitations. The finding of this study cannot be generalised to all health workers working at the included facilities because of likely self-selection bias. Equally selection with regard to health facilities is highly likely because facilities were selected on the basis of accessibility and logistics. Hence more decentralised and rural health facilities were not included in the study, which means that results cannot be generalised to these settings. In addition, social desirability bias may have influenced the results of the process evaluation. Health workers may not have felt free to discuss challenges encountered when accessing the health service.*

Journal Requirements:

2. Please review your reference list to ensure that it is complete and correct. If you have cited papers that have been retracted, please include the rationale for doing so in the manuscript text, or remove these references and replace them with relevant current references. Any changes to the reference list should be mentioned in the rebuttal letter that accompanies your revised manuscript. If you need to cite a retracted article, indicate the article’s retracted status in the References list and also include a citation and full reference for the retraction notice.

*Noted with thanks.*

3. We do not publish any copyright or trademark symbols that usually accompany proprietary names, eg  ©, ®, ™  (e.g. next to drug or reagent names). Please remove all instances of trademark/copyright symbols throughout the text, including ® on page 12.

*Trademark symbols have been removed. Thank you.*

4. Potential Copyright Issues:

Fig 3: please (a) provide a direct link to the base layer of the map (i.e., the country or region border shape) and ensure this is also included in the figure legend; and (b) provide a link to the terms of use/license information for the base layer image or shapefile. We cannot publish proprietary or copyrighted maps (e.g. Google Maps, Mapquest) and the terms of use for your map base layer must be compatible with our CC-BY 4.0 license.

*Thank you for your comment and in-depth clarification.*

*We have added a figure legend with the link to the source of the base maps on lines 293-295. It now reads:*

*“**The map was created using shape files from* [*https://data.humdata.org/dataset/cod-ab-zwe*](https://data.humdata.org/dataset/cod-ab-zwe) *(Humanitarian Data Exchange; Zimbabwe National Statistics Agency) and plotted in R using the sf and tmap packages.”*

*We used different openly available base maps (shape files) form the above link to create figures specifically for this publication and the figures were not used elsewhere or reproduced. License details are found on:* [*https://data.humdata.org/dataset/cod-ab-zwe*](https://data.humdata.org/dataset/cod-ab-zwe) *and* [*https://data.humdata.org/faqs/licenses*](https://data.humdata.org/faqs/licenses)*.*
